# Supplementary material for: Psychology at the screen: Investigating the current VAR protocol
Source: PLoS One. 2026 Apr 15;21(4):e0345704. doi: 10.1371/journal.pone.0345704 (PMC13082606; doi:10.1371/journal.pone.0345704)
Supplement: Attachment — Submitted filename: Responses_and_Amendments (1).docx [file pone.0345704.s002.docx]

Manuscript ID PONE-D-25-60174 titled “Psychology at the screen: Investigating the current VAR protocol”

**PLOS ONE**

The authors have attempted to address the reviewer’s suggestions as highlighted below.

| **Reviewer 1 comments to Authors** | | |
| --- | --- | --- |
| **Comment/Recommendation** | **Amendment** | **Location** |
| The results were thoroughly analyzed, and the data interpretation was performed accurately and cautiously. The additional details (following the initial review) are adequately meet expectations.  Furthermore, this article demonstrates sufficient scientific rigor for publication in the journal.  In conclusion, this article demonstrates sound scientific quality and is suitable for publication as is. | Thank you for these comments and for the time taken to review our work. | - |
| **Reviewer 2 comments to Authors** |  |  |
| **Comment/Recommendation** | **Amendment** | **Location** |
| Thank you for writing this manuscript. It is indeed very beneficial for research in the area of VAR in football, particularly the English Premier League (EPL). I do have some questions that I would like further clarification: | Thank you for these comments and for the time taken to review our work. | - |
| Line 73: How is match-changing incidents conceptually defined? | Thanks for this comment. We have now included in brackets the definition of what is meant by match-changing incidents. | L73-74 |
| Line 116-130: Are any of these research referring specifically to the English Premier League? It does not indicate which league, only a general mention of the countries. Since this study is specific to the (EPL), you might want to specify which studies are referring to the EPL. | Thanks for this comment. So, Pollard (2006; 2008) works are based on a wide range of countries – one referring to over 72 nations.  Carmichael & Thomas (2005) specifically refers to English Premier League.  Nevill et al. (1996) refers to top 8 divisions across England and Scotland (of course including EPL).  Seckin & Pollard (2008) measure Turkish Super League but comparisons are drawn with EPL.  Unkelbach & Memmert (2010) measure Bundesliga but draw comparisons with EPL.  We therefore believe that the work cited here is relevant to the EPL.  Additionally, we have made it clearer to the reader the specific divisions that Sors et al. (2022) reviewed. | L. 128-130 |
| Line 237-259: You might want to add the percentage (%) as well for the description of statistics to make it more reader friendly. For example: Of the 245 remaining decisions that were made, 148 (60.4%) were a decision that was directly related to the home team. | Great suggestion! We have now added percentages for other descriptive statistics to support reader’s interpretation. | L239-263 |
| Future Directions: What do you think about qualitative research, interviewing the experience of referees themselves, of whether they think external factors might influence their decisions? This comes into light after Mike Dean admitted on national television that his decision was influenced by being "mates" with other referees. | This is something I have considered and have been in communication with PGMOL over the 18 months of this project.  Unfortunately, the members of PGMOL I have been in contact with have been dismissive of this work – suggesting that their officials would make the right decision either way (obviously they are supporting their co-workers but the point being that we are all influenced implicitly).  In any case, yes I have considered this, and hopefully publishing this manuscript will help push this further.  I have added a sentence suggesting this as a future direction. | L402-404 |
